# Supplementary figures and images for: Immune activity score to assess the prognosis, immunotherapy and chemotherapy response in gastric cancer and experimental validation
Source: PeerJ. 2023 Nov 14;11:e16317. doi: 10.7717/peerj.16317 (PMC10655707; doi:10.7717/peerj.16317)

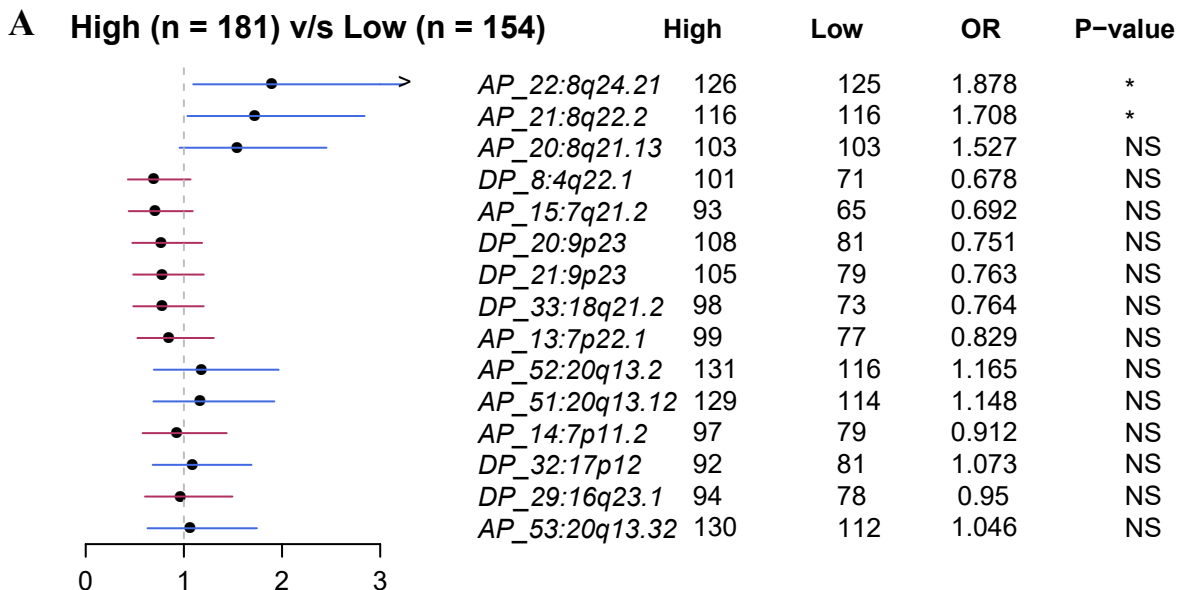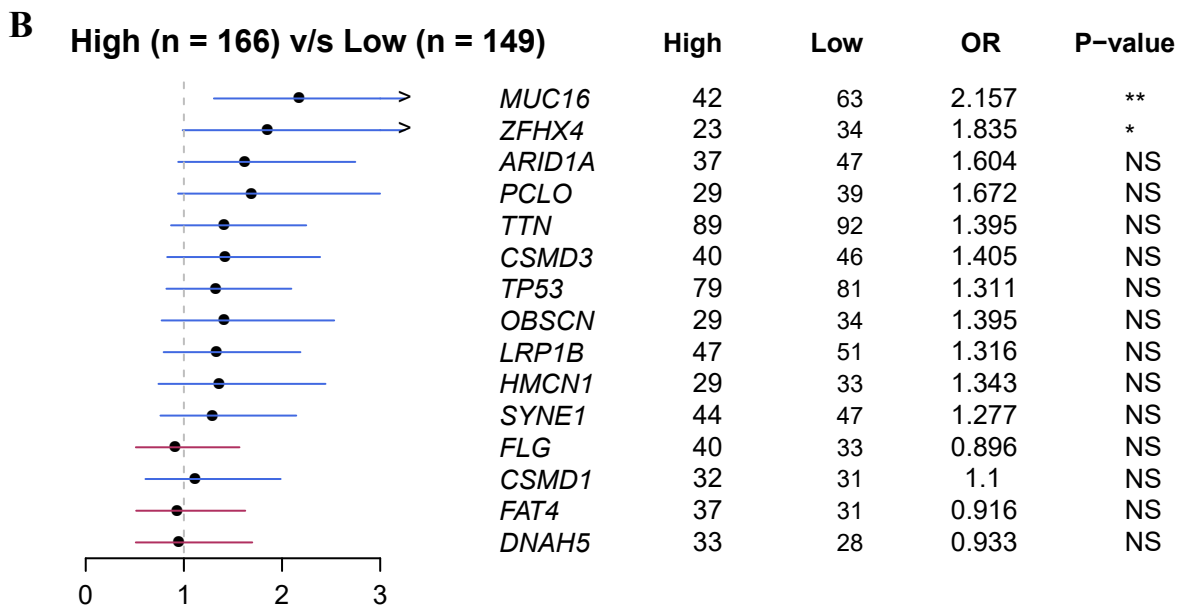

Supplement: Figure S1 — A, CNV mutation sites. B, Top 15 genes in terms of mutation frequency. [file peerj-11-16317-s001.pdf]

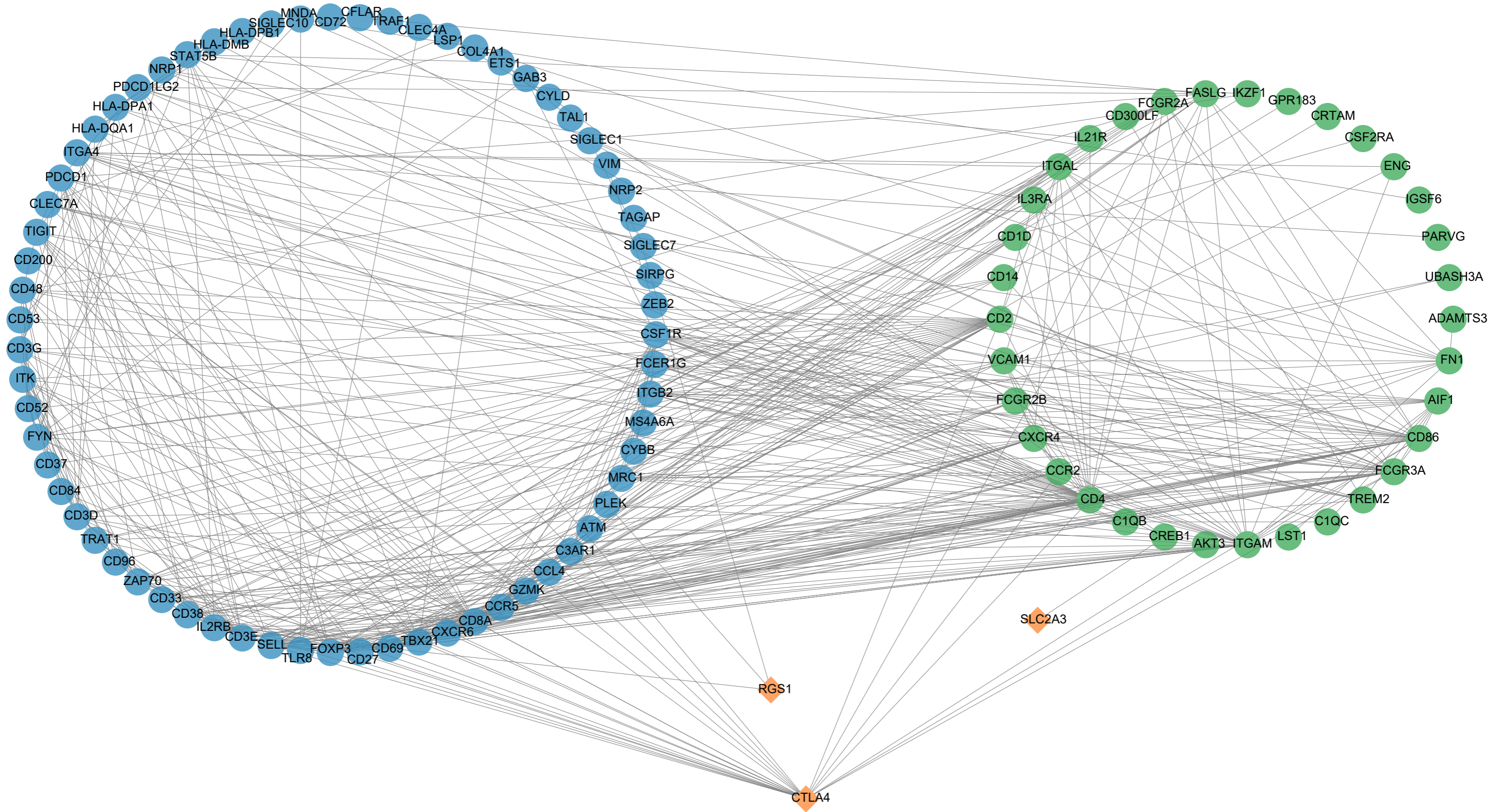

Supplement: Figure S2 [file peerj-11-16317-s002.pdf]

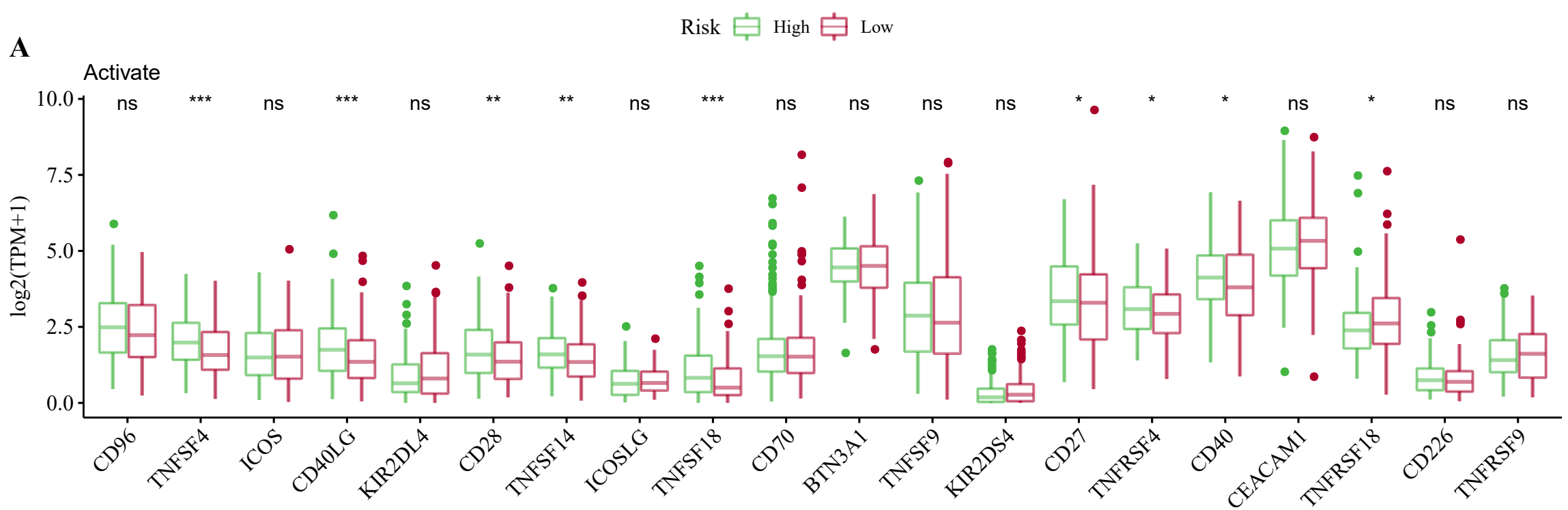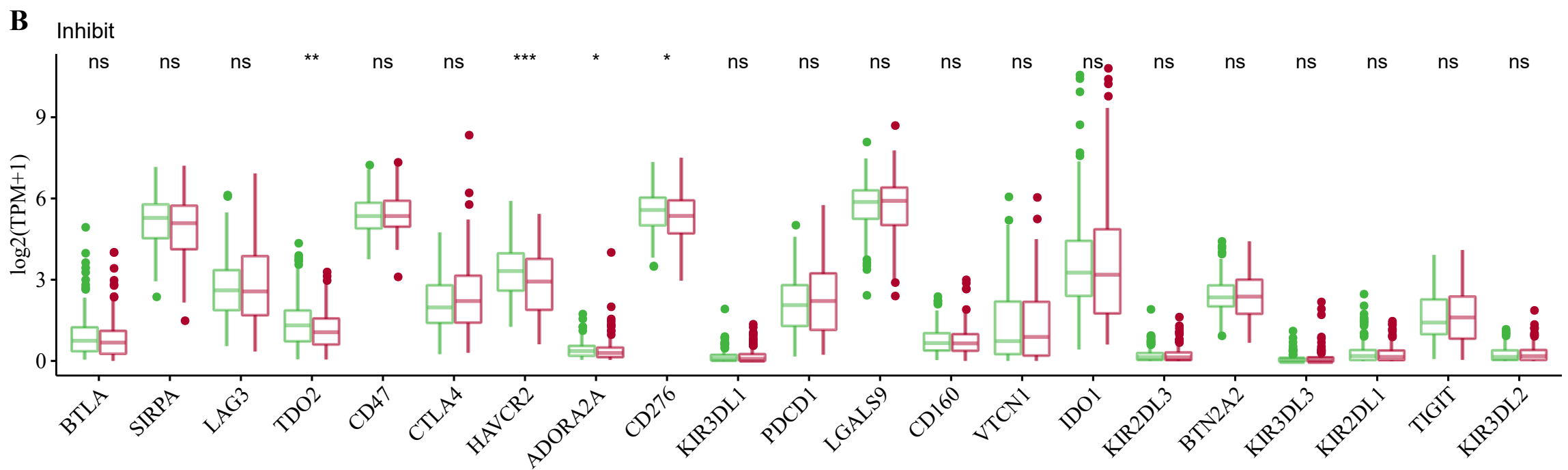

Supplement: Figure S3 — A: Expression levels of activate-related immune checkpoint genes in the high IAS and low IAS groups. B: Expression levels of inhibit-related immune checkpoint genes in the high IAS and low IAS groups. [file peerj-11-16317-s003.pdf]
